# A Prospective Study on the Predictability of Propofol Injection Pain

**DOI:** 10.7759/cureus.6945

**Published:** 2020-02-10

**Authors:** Omer Tasargol

**Affiliations:** 1 Anesthesiology, Dr. Burhan Nalbantoglu State Hospital, Nicosia, CYP

**Keywords:** neutrophil, propofol, pain

## Abstract

Introduction

In this prospective study, we aimed to determine if there was a link between pain on propofol injection (POPI) and various hemogram parameters and ratios.

Methods

The study was designed to include 100 consecutive male patients undergoing surgery in a tertiary hospital in February 2017. Preoperatively collected data included patients’ age, weight, height, hemogram parameters (white blood cell (WBC) count, neutrophil count, lymphocyte count, platelet count, mean platelet volume (MPV), platelet distribution width (PDW), plateletcrit (PCT), hemoglobin, and hematocrit). All patients’ hemograms were performed using the same device. The neutrophil-lymphocyte ratio, platelet-lymphocyte ratio, and systemic immune inflammatory score (SII) were calculated from this data.

Patients received prior information that pain would be questioned during propofol injection. The pain was grouped between 0 and 3 (0 no pain, 1 mild pain, 2 moderate pain, 3 severe pain).

Patients were questioned by the anesthesiologist about their level of pain at five-second intervals until loss of consciousness occurred. The same anesthesiologist also used the McCririck and Hunter’s verbal rating score (VRS) to evaluate pain. The patients’ heart rate and blood pressure were also recorded after induction.

Results

An extremely significant correlation was found between the presence of POPI and neutrophil-lymphocyte ratio (NLR), platelet lymphocyte ratio (PLR) and SII ratios (p<0.001). All three parameters were found to be significant in predicting the presence of POPI. The higher area under the curve (AUC) was found for SII.

The cutoff value for SII’s prediction of POPI was calculated to be 479,000, with a sensitivity of 68% and a specificity of 68%. Patients with an NLR of >497000 had a 4.63 times higher risk of POPI (odds ratio: 4.63, 95% CI: 1.6714 - 12.7982).

Conclusion

Our study is the first to show that POPI can be predicted by using NLR, PLR, and SII. Our data also support other studies that have reported a relationship between the inflammatory biomarker NLR and pain perception.

## Introduction

Propofol is the most frequently used hypnotic agent in anesthesia induction and total intravenous anesthesia (TIVA) [1]. Pain on propofol injection (POPI) is a commonly encountered clinical problem [1-3]. Many medications, such as lidocaine, fentanyl, and ephedrine, have been evaluated for the management of POPI [4-7]. The cause of pain during injection of propofol is theorized to be due to endothelial damage, osmolality difference, non-physiological pH, and stimulation of venous nociceptive receptors and free nerve ends although it is generally accepted to be multifactorial [2-8]. The incidence of POPI is reported to be between 28% and 90% and is considered to leave a bad memory of general anesthesia in patients [1].

Despite many studies on the management of POPI, data are scarce with regards to any correlation with patient demographics and the ability to predict POPI [9].

In recent years, studies have demonstrated a link between some values and ratios obtained from a hemogram and the diagnosis, follow-up, or survival rates of certain pathologies [10]. The neutrophil-lymphocyte ratio has been shown to have a relationship with the perception of postoperative pain and pain in chronic diseases [11-14].

In this prospective study, we aimed to determine if there was a link between POPI and various hemogram ratios (neutrophil-lymphocyte ratio (NLR), platelet lymphocyte ratio (PLR), systemic immune inflammation score (SII).

## Materials and methods

The study was designed to include 100 consecutive male patients undergoing surgery in a tertiary hospital in February 2017. Informed consent was obtained from all patients. Male patients classified as American Anesthesiology Association (ASA) physiological score I or II, aged between 18 and 40 years due to undergo general anesthesia for elective orthopedic, abdominal, urological, ear-nose-throat, or plastic surgery procedures were included in this study. Subjects with chronic diseases, such as diabetes, hypertension, hypo or hyperthyroidism, vitamin B12 or vitamin D deficiency, leucocytosis, leucopenia, or other hematological, biochemical, or serological abnormalities, those with chronic medication use, and those using non-steroid anti-inflammatory drugs in the previous week, steroid use in the previous six months (including steroid creams), those with upper respiratory tract infections within the last three weeks, and those with routine alcohol intake were excluded from the study, as these conditions may affect the results of hemogram parameters. Patients with a history of psychiatric illness and those who required the use of a sedative due to serious preoperative anxiety were also excluded, as these patients’ perception of pain may have been affected.

Preoperatively collected data were patients’ age, weight, height, hemogram parameters (white blood cell count, neutrophil count, lymphocyte count, platelet count, mean platelet volume (MPV), platelet distribution width (PDW), plateletcrit (PCT), hemoglobin, and hematocrit). All patients’ hemograms were performed using the same device (Sysmex XT 1800i, Sysmex Corporation, Kobe, Hyogo, Japan). NLR, PLR, and SII were calculated from this data. We calculated SII from the equation, SII = Platelet (P) x Neutrophil (N) / Lymphocyte (L), where P, N, and L are the preoperative peripheral blood platelet and neutrophil and lymphocyte counts per liter, respectively.

Patients underwent routine monitoring (including electrocardiography, pulse oximetry, and noninvasive blood pressure). Intravenous access was performed on the dorsum of the hand with a 20 gauge cannula. A face mask was used to deliver 6 lt/min of fresh oxygen and fraction of inspired oxygen (FiO2) for three minutes before induction.

Patients received prior information that pain would be questioned during propofol injection. The anesthesiologist used the McCririck and Hunter’s verbal rating score (VRS) to evaluate pain. Pain was grouped between 0 and 3 (0 no pain, 1 mild pain, 2 moderate pain, 3 severe pain). A solution of 200 mg/20 mL 1% propofol and 2 mL 2% lidocaine (40 mg) was prepared in a 50 mL injector. Basal heart rate and blood pressure were measured and propofol infusion of 18.3 mL/min was commenced and continued until a dose of 2.5 mg/kg was achieved. The patients were questioned by the anesthesiologist about their level of pain at five-second intervals until loss of consciousness occurred. The patients’ heart rate and blood pressure were also recorded after induction.

A blinded individual compared hemogram parameters and ratios to pain perception scores. SPSS 16.0 (SPSS, Chicago, IL, USA) was used for statistical analysis. Parameters were compared using the independent samples t-test for normal distribution; otherwise, the Mann-Whitney U test. An intergroup comparison was performed using the analysis of variance (ANOVA) test. Cut-off levels for parameters (sensitivity and specificity) were calculated using the receiver operating characteristic (ROC) curve analysis. p <0.05 was regarded as statistically significant.

## Results

Of 100 patients, eight were excluded due to leukocytosis, thrombocytopenia, and other hematological problems, seven due to chronic alcohol use, five due to psychiatric pathologies, and one due to preoperatively detected arrhythmia. The average age of the remaining 79 patients was 35.1±7.8 years and the average body mass index was 26.04±2.81.

When POPI was evaluated using VRS, 25 patients had no pain, 31 had mild pain, 14 had moderate pain, and nine had severe pain. The patients’ distribution of VRS scores, demographics, and hemogram parameters are shown in Table 1. The comparison of data in patients with or without POPI is shown in Table 2.

**Table 1:** Demographic and hemogram parameters for patients grouped according to pain intensity Body Mass Index: BMI; White Blood Cell: WBC; Hemoglobin: HGB; Hematocrit: HCT; Platelet: PLT; Platelet Distribution Width: PDW; Mean Platelet Volume: MPV; Plateletcrit: PCT; Neutrophil Count: NEUT#; Lymphocyte Count: LYMPH#; Neutrophil Lymphocyte Ratio: NLR; Platelet Lymphocyte Ratio: PLR; Standard Derivation: SD; McCririck and Hunter’s Verbal Rating Score: VRS; Systemic Immune Inflammation Score: SII; Number of Individuals: n

	VRS:0 n=25		VRS:1 n=31		VRS:2 n=14		VRS:3 n=9		All Patients n=79	
	Mean	SD	Mean	SD	Mean	SD	Mean	SD	Mean	SD
BMI	25.50	2.94	26.53	2.79	26.91	2.46	24.52	2.52	26.04	2.81
WBC	7.75	2.38	9.09	2.25	7.92	2.61	9.52	2.85	8.51	2.48
HGB	12.68	3.14	13.84	1.91	14.56	1.82	14.82	1.27	13.71	2.40
HCT	39.33	6.44	41.70	5.35	43.13	4.73	43.98	3.45	41.46	5,61
PLT	239.24	86.82	298.32	109.20	265.00	42.40	412.22	175.97	286.70	114.28
PDW	12.26	2.10	11.74	1.91	12.15	1.16	11.82	1.59	11.99	1.82
MPV	10.36	0.78	10.15	0.95	10.24	0.70	10.20	0.69	10.24	0.82
PCT	0.24	0.09	0.29	0.09	0.27	0.04	0.39	0.19	0.28	0.11
NEUT#	3.92	1.73	5.30	1.76	4.93	1.99	6.07	2.17	4.88	1.95
LYMPH#	2.14	0.86	2.35	0.84	2.55	0.81	2.20	0.62	2.30	0.82
NLR	1.84	0.74	2.87	2.14	2.13	1.19	2.97	1.34	2.43	1.61
PLR	119.52	37.86	138.24	63.91	110.04	23.69	217.70	150.56	136.37	73.56
SII	476.12	289.92	762.06	547.14	542.45	254.47	1392.73	1275.30	704.50	629.29

**Table 2:** Comparison of demographic and hemogram parameters for patients according to the presence or absence of pain Body Mass Index: BMI; White Blood Cell: WBC; Hemoglobin: HGB; Hematocrit: HCT; Platelet: PLT; Platelet Distribution Width: PDW; Mean Platelet Volume: MPV; Plateletcrit: PCT; Neutrophil Count: NEUT#; Lymphocyte Count: LYMPH#; Neutrophil Lymphocyte Ratio: NLR; Platelet Lymphocyte Ratio: PLR; Standard Derivation: SD; McCririck and Hunter’s verbal rating score: VRS; Systemic Immune Inflammation Score: SII; Number of Individuals: n; 95% Confidence Interval: p

	VRS:0 n=25		VRS:1-2-3 n=54		
	Mean	SD	Mean	SD	p
BMI	25.50	2.94	26.29	2.74	0.655
WBC	7.75	2.38	8.85	2.47	0.863
HGB	12.68	3.14	14.18	1.81	0.302
HCT	39.33	6.44	42.45	4.92	0.104
PLT	239.24	86.82	308.24	104.23	0.301
PDW	12.26	2.10	11.85	1.67	0.166
MPV	10.36	0.78	10.18	0.83	0.758
PCT	0.24	0.09	0.29	0.10	0.580
NEUT#	3.92	1.73	5.33	1.88	0.670
LYMPH#	2.14	0.86	2.37	0.797022	0.590
NLR	1.84	0.74	2.69	1.82	<0.001
PLR	119.52	37.86	150.59	94.30	<0.001
SII	476.12	289.92	837.07	725.74	<0.001

**Figure 1:**
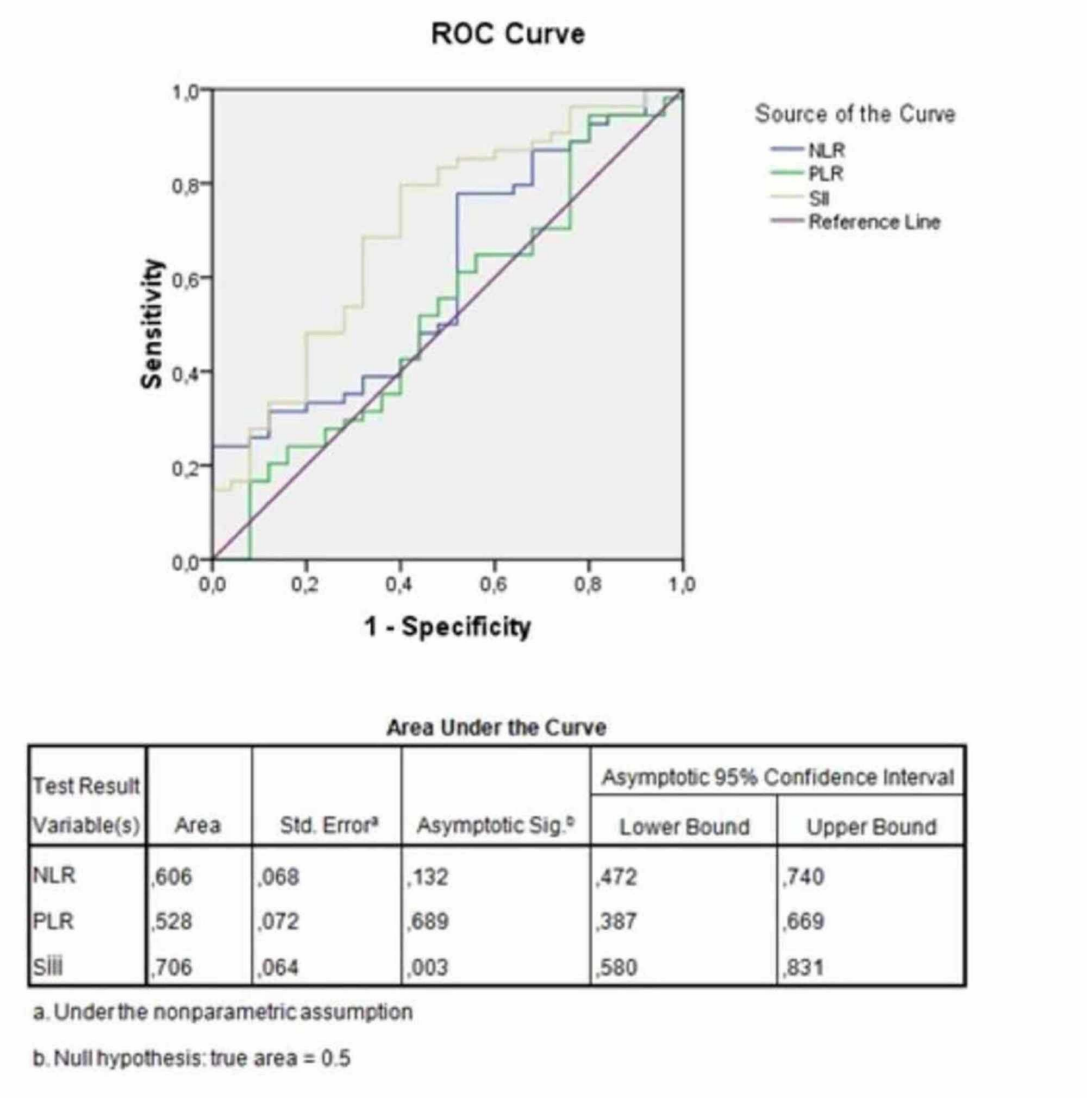
ROC curve for NLR, PLR, and SII Neutrophil Lymphocyte Ratio: NLR; Platelet Lymphocyte Ratio: PLR; Systemic Inflammation-Immune Ratio: SII; Receiver Operating Characteristic: ROC

An extremely significant correlation was found between the presence of POPI and NLR, PLR, and SII ratios (p<0.001). Area under the curve (AUC) for NLR, PLR, and SII in regards to the presence of POPI is shown in Figure 1. All three parameters were found to be significant in predicting the presence of POPI. The higher AUC was found for SII.

The cutoff value for NLR ‘s prediction of POPI was calculated to be 1.64, with a sensitivity of 77% and specificity of 48%. Patients with NLR of >1.64 had a 3.23 times higher risk of POPI (odds ratio: 3.23, 95% CI: 1.1726 - 8.9018).

The cutoff value for PLR‘s prediction of POPI was calculated to be 121000, with a sensitivity of 56% and a specificity of 52%. Patients with NLR of >121000 had a 1.46 times higher risk of POPI (odds ratio: 1.46, 95% CI: 0.5635 - 3.7836).

The cutoff value for the SII prediction of POPI was calculated to be 479000, with a sensitivity of 68% and a specificity of 68%. Patients with an SII of >497000 had a 4.63 times higher risk of POPI (odds ratio: 4.63, 95% CI: 1.6714 - 12.7982).

## Discussion

While our study did not find a correlation between any individual hemogram parameter and POPI, three ratios calculated from these parameters (NLR, PLR, and SII) were found to be significantly higher in patients with POPI.

POPI is a clinical problem that must be managed by anesthesiologists. Literature reports that POPI can be seen in up to 90% of patients, especially when the injection is made to small diameter veins such as those on the dorsum of the hand [1,15]. Many agents, such as lidocaine, ketamine, ephedrine, and magnesium, have been used for the prevention of POPI [2,16-17]. However, the most common application in clinical settings is the application of lidocaine before propofol or an admixture of propofol with lidocaine [18-19]. In our study, we tried to prevent POPI by adding 20 mg of lidocaine to 200 mg of propofol.

Hanci et al. reported that POPI differed at different times of the menstrual cycle in women [9]. They observed that POPI was more frequently seen in the luteal phase. We are unaware of any other study that predicted POPI.

NLR, PLR, and SII are cheaply and easily available biomarkers calculated from hemogram parameters that have been shown to be useful in the differential diagnosis of various pathologies as well as predictors of disease survey and treatment response [10,13,20]. These biomarkers associated with acute or chronic inflammation have also been reported to be related to the severity and perception of acute and chronic pain [13-14]. In our study, we have determined that high NLR, PLR, and SII are associated with POPI and its perception.

In a study of ASA I-II patients due to undergo laparoscopic cholecystectomy, Persson et al. evaluated the relationship between postoperative pain intensity measured using VAS and opioid consumption during venous cannulation and propofol injection [21]. The authors found a positive correlation between both the VAS score during propofol injection and postoperative pain intensity and the VAS score during venous cannulation and opioid consumption. We did not evaluate postoperative pain intensity in our study. However, we have demonstrated that NLR, PLR, and SII calculated from hemogram parameters can be used to predict POPI. It could be implied that these ratios and indexes could also be related to pain intensity. Further comprehensive studies may lead to a personalized postoperative pain regimen according to these preoperatively calculated ratios.

There are many limitations to this study. First, a study evaluating the relationship between NLR, PLR, and SII and smoking found that these ratios and indexes are significantly higher in smokers [22]. We did not exclude nonsmokers or smokers in our study. This may be considered a limitation. The menstrual cycle has been shown to change POPI and we, therefore, did not include female patients [9].

## Conclusions

Our study is the first report to show that POPI can be predicted by using NLR, PLR, and SII. Our data also support other studies that have reported a relationship between the inflammatory biomarker NLR and pain perception.
